# Exploring Integration of Care for Children Living with Complex Care Needs across the European Union and European Economic Area

**DOI:** 10.5334/ijic.2544

**Published:** 2017-04-24

**Authors:** Maria Brenner, Miriam O’Shea, Philip J Larkin, Stine Lundstroem Kamionka, Jay Berry, Harriet Hiscock, Michael Rigby, Mitch Blair

**Affiliations:** 1 School of Nursing and Midwifery, Trinity College Dublin, 24 D’Olier St, Dublin 2, IE; 2 School of Nursing, Midwifery and Health Systems, University College Dublin and Our Lady’s Hospice and Care Services, Dublin, IE; 3 Centre for Global Health, University of Southern Denmark, Odense, DK; 4 Department of Medicine and Division of General Pediatrics, Boston Children’s Hospital and Harvard Medical School, Boston, MA, US; 5 Murdoch Children’s Research Institute, Centre for Community Child Health, Royal Children’s Hospital, Parkville VIC 3052, AU; 6 Section of Paediatrics Faculty of Medicine, Imperial College of Science, Technology and Medicine, GB

**Keywords:** child health, europe, models, vignettes

## Abstract

**Introduction:**

The aim of this paper is to report on the development of surveys to explore integration of care for children living with complex care needs across the European Union (EU) and European Economic Area (EEA).

**Theory and methods:**

Each survey consists of a vignette and questions adapted from the *Standards for Systems of Care for Children and Youth with Special Health Care Needs* and the *Eurobarometer Survey*. A Country Agent in each country, a local expert in child health services, will obtain data from indigenous sources.

**Results:**

We identified ‘in-principle’ complex problems and adapted surveys to capture care integration. We expect to get rich data to understand perceptions and to inform actions for a number of complex health issues.

**Conclusion:**

The study has the potential to make a wide contribution to individual countries of the EU/EEA to understand their own integration of services mapped against responses from other member states. Early results are expected in Spring 2017.

## Background

The United Nations Convention on the Rights of the Child (to which all European Union (EU) and European Economic Area (EEA) Member States are signatories) defines the highest attainable standard of healthcare as a fundamental right of every child [1]. The extent to which this requirement is met in practice by national healthcare systems, varies considerably among the countries of Europe, and is the core purpose of the Horizon 2020 funded project Models of Child Health Appraised (MOCHA), running from 2015 to 2018 [2]. This study is embedded in the various peculiarities of national healthcare systems and the ethical and legal concerns bound to the sharing of child health data. One aspect of the MOCHA project is to provide an updated comprehensive analysis of the current approach in each EU and EEA Member State to managing the care of children living with complex care needs, with particular regard to the integration of care at the acute/community/primary interface. Integrated care refers to the management and delivery of health services so that children and their families receive a continuum of preventive and curative services, according to their needs over time and across different levels of the health system [3].

It is imperative to address the issue of integrated care for a growing population of children living with complex health issues. Improvements in neonatal and paediatric care mean that more children with complex care needs are surviving into adulthood. By their very nature children, and families of children, with complex care needs place great challenges on healthcare delivery in the community [4]. While a relatively small proportion of the population, the cost of healthcare and support services for this group is very high; figures from the United States show that children with complex health needs account for as much as one-third of healthcare spending for all children [5]. Although the provision of care closer to home for such children is a policy objective internationally [6], integration of health services is insufficient with wide variation in systems of care for these children internationally. Progress towards achievement of this goal has been slow despite growing evidence that homecare: provides a means of mitigating the barriers and isolation children and their families experience during the transition from hospital to home, can significantly reduce hospital utilisation, and reduces the cost of care for children living with complex care needs [7,8].

This paper reports on the development of the vignettes and adaptation of surveys to explore the integration of care services for this population across the EU and EEA, to understand the influences on the integration of healthcare services and to explore the context for the answers provided within each country. We present some of the particular challenges and benefits of using case vignettes in a large EU study.

## Theory and Methods

The challenge was to develop a research approach that could help facilitate comparative research by providing a data collection method that could be used across 30 states. The methods used were influenced by the current move to use both quantitative and qualitative approaches in the exploration of structures and processes of care provision. This is a non-experimental descriptive study with a qualitative element; the decision to use a mixed-method approach was based on a pragmatic and pluralist approach, informed by discussion on post-positivism advocating a realist perspective on healthcare research [9].

### Vignette and survey development

The criteria for selection of areas for study were steered by the following: consideration of previous work in this area including trends of presentation and findings on burden of care; completion of systematic and integrative reviews; exploring care at a variety of ages between infant and 18 years of age; and congruity with other ongoing work across the wider MOCHA project. Five vignettes were developed for children requiring integrated care in each of the following areas: long-term ventilation, intractable epilepsy, traumatic brain injury, attention deficit hyperactivity disorder and autism. The decision on the specific areas to be explored was initially made by a team comprised of clinical and academic expertise including senior nursing academics with backgrounds in critical and palliative care, and physicians in complex and community care. These areas were subsequently ratified by the External Advisory Board to the project which is comprised of European medical, paediatric and policy bodies, and civil society groups including a young person from the youth sub-group of the European Patients’ Forum.

Each survey consists of three sections – a vignette, questions adapted from the *Standards for Systems of Care for Children and Youth with Special Health Care Needs* (CYSCHN) [10] and the Complex Care European Survey of Change, adapted from the Eurobarometer Survey [11]. The survey will be applied in each of the 30 study countries, using a local agent. It therefore needed to be clear, with terms and constructs that would be unambiguous independently of any local structures, or practice styles or conventions.

Vignette development and survey adaptation was guided by a rights-based framework, in that our work was informed by the work of the UN Convention on the Rights of the Child [1] and guided by the following principles: universality and inalienability; indivisibility; interdependence and interrelatedness; equity and non-discrimination; participation and inclusion; empowerment; accountability and respect for the rule of the law. This work was also informed by philosophy of family-centered care, which suggests that the care of a child is best delivered in consultation with the child and their family [12]. The central tenet of this philosophy of care is that optimum care of a child is achieved through a partnership approach with the child’s family. In theory, this recognizes the uniqueness of each family and builds on the strengths of each family, though it is widely acknowledged that policy makers often omit meaningful reference to family engagement and healthcare professionals often struggle to achieve family-centered care in practice [13].

#### Writing the vignettes

In the past vignettes were predominantly used in politics and marketing, however, they have recently been used for a variety of reasons in healthcare research including patient preferences in shared decision making [14] and practitioner assessments of parenting [15]. This approach is also compatible with the innovations of Yin in using case studies in health services research [16]. Guided by best practice in writing vignettes [17,18,19] the research team drew on their own clinical expertise, findings from previous studies exploring the coordination and integration of care for children living with complex health needs and an extensive search of the literature. This was to ensure that the vignettes would contain sufficient clinically relevant information on the setting, the participants, the problem and the interacting dimensions, to allow participants the clearest possible picture of the situation.

In writing the vignettes for use in 30 countries we were mindful of the need for clarity in terminology, the need to present a setting that could be widely understood, and consideration of the optimum length of the vignettes. The issue of language was particularly important as the official language of the MOCHA project is English and there was no opportunity for translation and back translation within the project (the MOCHA Country Agents having been selected as being adequately fluent in scientific English as well as the country’s indigenous language(s)). To address the issue of terminology we developed a glossary of terms to accompany each survey. This was part of an overall glossary of terms for the work package, which was also available on the project website. A choice had to be made regarding whether the vignette was to be a “snapshot” (a static situation) or representing a process with different stages [20]. In keeping with best practice the vignettes are of a moderate length, no longer than one paragraph, and reflect a static situation, to avoid over-burden on the respondents [15,21]. An example of the vignette used to explore the integration of services for long-term ventilation is presented here:

Max is an eighteen month old boy with a diagnosis of chronic lung disease due to bronchopulmonary dysplasia. Max was born at 26 weeks gestation weighing less than 1 kg. He had a diaphragmatic hernia, a gastrostomy tube placement at three months of age, and a Grade IV intraventricular hemorrhage requiring a cerebrospinal fluid ventricular shunt. Max has been ventilator dependent since he was born and is considered to have a life-threatening condition. A tracheostomy tube was placed at six weeks of age due to the need for ongoing ventilation. Max spent the first three months of his life in intensive care, followed by four months in a step-down/transitional care unit. At present Max has the following: impaired pulmonary function, developmental delay in fine and gross motor skills, and speech and language difficulties. His prognosis for weaning off the ventilator does not seem favorable at the moment and ideally he requires the healthcare input of the following healthcare professionals: community nurses, specialist consultants (respiratory, pediatrician, neurology), community general practitioner, pharmacist, speech and language therapist, physiotherapist, occupational therapist, social worker, dentist, homecare nursing team and respite care services. He lives with his two sisters, aged 5 and 7 years, and his mother and father. He lives 120 kms from the main children’s hospital and 40 kms from his nearest regional hospital which has a small pediatric unit.

#### Identification of survey instruments

An extensive review of the literature identified a number of potential instruments, however, the majority of these tools were focused very specifically on care coordination practices as opposed to seeking to explore the integration of care of children living with complex care needs at the acute/community/primary interface. We therefore made the decision, with permission of the Lucile Packard Foundation, to adapt the *Standards for Systems of Care for Children and Youth with Special Health Care Needs (CYSHCN)* [10]. These standards address the core components of the structure and process of an effective system of care for this population. They were initially derived from a comprehensive review of the literature, expert opinion, and case studies of standards currently in use across the US with input and guidance from a national work group of stakeholder including pediatric providers, health plans, children’s hospitals, families/consumers, health services researchers, and others.

The final part of the survey focuses on gathering data on the socio-cultural context for the responses given for each of the States. The *Complex Care European Survey of Change* was adapted from a specific Eurobarometer Survey [11]. This section of the data collection tool includes questions on barriers to, and opportunities for the management of children living with complex care needs, questions on policy, and questions on the future of care delivery at the acute community interface to children living with complex care needs in each country.

### Sample

The vignettes and surveys were delivered to a Country Agent in each of the 30 countries. This is a key methodological feature of the MOCHA project, the remunerated retention in each study country of a part-time Country Agent – a local expert in child health services – who acts as the informant for obtaining data requested by the principal scientists in the project, from local indigenous sources.

### Validity and reliability

Although face validity does not provide strong evidence of validity, it is a helpful means of encouraging participation in the study [22]. Face validity was established through consultation with other researchers and clinical experts to determine professional appearance and layout. To address content validity the vignettes were sent to two experts who had experience in vignette development. The vignettes and surveys were also presented and discussed with a large group of stakeholders including: clinical experts in acute and community settings; healthcare managers and discharge coordinators; a number of European patient advocacy groups including the European Association of Children in Hospital, the European Patient Forum Youth Group and the European Association of Palliative Care; and other MOCHA researchers who would subsequently require the results of our work to progress modelling of processes of care. Qualitative measures of rigor (credibility, authenticity, accuracy, confirmability and transferability) will be applied to the data [23].

### Considering the analytical phase

Data will initially be analyzed using descriptive statistics; frequency and frequency percent will also be reported. The qualitative data analysis software program NVivo 10 will be used to support data management [24]. Significant codes will initially be identified and then clustered to produce themes.

## Results

The use of vignettes affords the possibility to create a variety of care delivery situations pertaining to complex care. However, there is concern about the use of hypothetical situations to elicit opinions. This is considered a threat to external validity in the study as there is the potential that participants’ responses will not reflect the reality of clinical work. This reflects concerns raised in previous studies [17,25]. It has also been suggested that visual representation of a scenario would be better retained by the participants and could capture more of the nuances of real life [26]. However, the type of scenarios required for this study would have been quite difficult to depict visually. To address these concerns the vignettes were reviewed by a wide variety of experts mentioned above. The ‘complexity’ of measuring the structures and processes of complex care across 30 countries cannot be underestimated. We expect that the vignettes developed will achieve a reasonable level in the standardization of data collection across 30 countries. In the development of the vignettes we consider that we have identified ‘in-principle’ complex problems and that the surveys will capture how they are dealt with. We therefore expect that the comparative potential is high across different countries. We are also optimistic that we will get rich contextual data that will be crucial for making sense of perceptions, beliefs, judgements and suggested actions for a number of complex health issues in childhood.

## Conclusion

The outcome of the study has the potential to make a wide contribution to the care of children living with complex health conditions across the EU/EEA. Early results are expected in Spring 2017. Further work, using the same surveys will be used within the project to map the EU/EAA picture against that in the United States for children on long-term ventilation and against that in Australia for children with enduring mental health conditions.
